# The burden of risk factors for non-communicable disease in rural Bihar, India: a comparative study with national health surveys

**DOI:** 10.1186/s12889-022-13818-1

**Published:** 2022-08-12

**Authors:** Stephanie Ross, Kashika Chadha, Shantanu Mishra, Sarah Lewington, Sasha Shepperd, Toral Gathani, on behalf of the NCDRI study collaborators, Sandra Albert, Apoorva Bhatnagar, Kashika Chadha, Toral Gathani, Ben Lacey, Sarah Lewington, Shantanu Mishra, Jennifer Roest, Stephanie Ross, Sasha Shepperd, Mara Violata, Sanjay Gupta, Vivek Singh, Rajiv Sarkar

**Affiliations:** 1 Nuffield Department of Population Health (NDPH), University of Oxford, Oxford, UK; 2 Dwhani Rural Information Systems, New Delhi, India; 3 Oxford Policy Management, New Delhi, India; 4 Oxford University Hospitals NHS Foundation Trust, Oxford, UK

**Keywords:** Non-communicable disease, Hypertension, Obesity, India National surveys

## Abstract

**Background:**

The incidence of non-communicable diseases (NCDs) is increasing in rural India. The National Family Health Survey-5 (NFHS-5) provides estimates of the burden of NCDs and their risk factors in women aged 15–49 and men aged 15–54 years. The aim of this study is to estimate the prevalence of hypertension and body-mass index (BMI) in adults aged 35–70 years in rural India and to compare these estimates, where age ranges overlap, to routinely available data.

**Methods:**

The Non-Communicable Disease in Rural India (NCDRI) Study was a cross-sectional household survey of 1005 women and 1025 men aged 35–70 conducted in Bihar in July 2019. Information was collected on personal characteristics, self-reported medical history and physical measurements (blood pressure, height and weight). Prevalence estimates for hypertension (systolic blood pressure ≥ 140 mmHg or diastolic blood pressure ≥ 90 mmHg, or diagnosed and treated for hypertension), and for underweight (body-mass index < 18.5 kg/m^2^), normal weight (18.5–25.0 kg/m^2^) and overweight (≥ 25.0 kg/m^2^) were calculated. Where age ranges overlapped, estimates from the NCDRI Study were compared to the NFHS-5 Survey.

**Results:**

In the NCDRI Study, the estimated prevalence of hypertension was 27.3% (*N* = 274) in women and 27.6% (*N* = 283) in men aged 35–70, which was three-times higher in women and over two-times higher in men than in the NFHS-5 Survey. One-quarter (23.5%; *N* = 236) of women and one-fifth (20.2%; *N* = 207) of men in the NCDRI Study were overweight, which was approximately 1.5 times higher than in the NFHS-5 Survey. However, where age groups overlapped, similar age-standardized estimates were obtained for hypertension and weight in both the NCDRI Study and the NFHS-5 Survey.

**Conclusion:**

The prevalence of NCDs in rural India is higher than previously reported due to the older demographic in our survey. Future routine national health surveys must widen the age range of participants to reflect the changing disease profile of rural India, and inform the planning of health services.

**Supplementary Information:**

The online version contains supplementary material available at 10.1186/s12889-022-13818-1.

## Introduction

The incidence of non-communicable diseases (NCDs) and their associated risk factors is increasing in all parts of India [1–4], as a consequence of the epidemiological transition over the last three decades. Rapid economic growth and subsequent development has resulted in a shift from the dominance of communicable (infectious) diseases to a rising burden of NCDs [5]. Although more developed states of India have observed an increase in NCDs for some time, significant increases are now being observed in rural states as well [6–8]. For example, in the state of Bihar (in the East of India), which has the lowest Human Development Index [9] and a population of 104 million projected to increase to 149 million in 2036[10], a higher burden of NCDs has been observed but the rate of increase is slower than in the more developed states [1]. However, much of the current literature about the state of health in rural areas of India, including Bihar, remains focused on communicable diseases.

Data from national surveys in India, including the India State-Level Burden Initiative from the Global Burden of Disease group [1, 5] and the National Family Health Survey (NFHS) [ 11–15], and the National Non-communicable Disease Monitoring Survey (NNMS) [16] inform policy makers on the current burden of disease. The NFHS has been routinely conducted in India since 1992 and is now in its fifth iteration (NFHS-5) [15]. The original ambition of the NFHS was to collect information on maternal and childhood health indicators, but the scope has now been widened to include a larger sampling frame and to collect additional information on risk factors that are associated with NCDs (e.g. height, weight, blood pressure and random fasting glucose). The NFHS surveys provide valuable insights into the health of rural India but there is a growing recognition that they may not be representative of the changing demographic profile of the country and therefore are not capturing the true patterns of disease burden [17]. The NFHS-5 Survey sampled women aged 15–49 and men aged 15–54 years, and therefore, cannot provide information about the prevalence of NCDs in older adults, who represent a growing proportion of the Indian population and in whom the incidence of NCDs and their associated risk factors is known to be higher [1, 5, 10, 18].

The aim of this study is to estimate the prevalence of hypertension and calculate body mass index (BMI), in a large cross-sectional household study of middle aged people in the Patna district of Bihar, a largely rural population, and where possible to use these data to compare to the NFHS-5 Survey estimates for the state.

## Methods

### Data sources

#### NCDRI study

The NCDRI Study was a population-based, cross-sectional household survey conducted in 2019. For administrative purposes, each state in India is divided into districts and blocks. Each block consists of groups of five to six villages known as gram panchayats. The study took place in two similar taluka/blocks among specified villages in the Patna district of Bihar. One block was used to pilot questionnaires and the second block was used for the full study.

The household questionnaire was adapted from questions previously validated in the World Health Organization (WHO) “STEPS methodology” [19], the NFHS-4 in India in 2015 [11] and the 2011 Census of India [20]. The questionnaires were developed and validated during a pilot study conducted in June 2019 using the SurveyCTO platform (Dobility, Inc; Cambridge, MA, USA). During the pilot study, a series of community engagement exercises were conducted to introduce the study to villages and to seek local permissions from community and village leaders, as well as representatives from the Ministry of Health. Feedback from these community engagement activities and from the interviewers involved in data collection in the pilot study, were used to inform the content and acceptability of the questionnaires, obtaining physical measurements from participants and electronic data collection using mobile tablets. Although measurement of blood pressure, weight and height were acceptable to community leaders and participants, the measurement of waist circumference was not and so this was not recorded.

Interviewers were recruited from selected local villages and were provided with two days of dedicated training in the local language by the study team. The training included instruction on the steps needed to take physical measurements of blood pressure, height and weight and the importance of using the same methods for each participant. Each interviewer was provided with a mobile tablet for data collection, a measuring tape, portable weighing scales and a sphygometer. Arrangements were made for the tablets to be charged on a daily basis by the study team so that no costs for electricity were transferred to the interviewers. All interviews were conducted in local dialects and all participants provided informed consent which was recorded electronically.

Participants were recruited into the main study during July 2019. In the villages selected for participation, community leaders provided maps of the village layout and routes for the interviewers to follow were agreed. The first household to be approached was identified and then every tenth household in the village was systematically approached for participation in the study. One eligible household member was invited to participate and this alternated between women and men to ensure deliberate equal representation. If a male member of the household was identified to be working in the fields near the village, interviewers were permitted to recruit and conduct interviews in the fields to avoid gender being a barrier to participation. Participants were eligible if they were a permanent resident of the household sampled (i.e. not a visitor/guest of the household from another locality), were aged 35–70 years inclusively and were able to provide proof of age (acceptable proofs of age included national identity cards, government issued ration cards, passport or driving licence). National identity cards were widely introduced in India in 2009, and as they are required to access Government benefits and services, > 95% of participants had one and this was the most common proof of age provided.

Information was collected on sociodemographic variables including age, sex, religion (Hindu, Muslim, other), education (no formal schooling, 1–4 years of schooling, 5–8 years or 9 years or more), occupation (housewife/hold, agricultural labour, self-employed/own business, other), household conditions (including access to running water, access to an indoor toilet), lifestyle factors (history of tobacco and alcohol use), and self-reported medical history for common NCDs, including a history of hypertension, and detail of any treatment.

Physical measurements were taken once at the time of the interview and included recorded measurements of height (cm), weight (kg) and blood pressure (mmHg). Every evening the study team collected the tablets from the interviewers so they could be charged and for the completed surveys to be uploaded onto the SurveyCTO platform. To ensure data quality, a random sample of 5% of participating households were re-surveyed within 24 h of interview (the timeframe was stipulated by the local ethics committee) using fifteen key pre-selected survey questions. The level of agreement for the responses obtained in the re-survey was very high and no concerns were identified about the quality of the initial data collection.

Ethical approval for the NCDRI study was obtained from the Local Institutional Ethics Review Board in India (Sigma Research and Consulting) and the Oxford Tropical Research Ethics Committee (OxTREC) at the University of Oxford.

#### National family health survey

The National Family Health Survey 2019–2020 (NFHS-5) is the fifth national, cross-sectional household survey conducted in India, and is co-ordinated by the International Institute for Population Sciences, Mumbai and data are publically available at state and district level [15]. Details of the sampling frame of all iterations of the NFHS are provided in Additional file: Supplementary Table 1. Full details of the study methodologies employed for the NFHS-5 are provided elsewhere [15]. Briefly, the NFHS-5 Survey employs a two-stage stratified sampling design and information on health and nutrition indicators and measurements of random blood glucose and standardized blood pressure are collected among urban and rural areas within each state. The NFHS-5 was conducted in 38 districts of Bihar between July 9th 2019 to February 2nd 2020 and information was collected from 35,834 households including 42,483 women aged 15–49 and 4897 men aged 15–54 years [21]. The state level reports are more granular than the district reports, with data reported by age and sex.

#### The 2011 census of India

In India, the national census is conducted every ten years. The 2011 Census of India has been used as baseline data to project the age compositions of the Indian population to the year 2036 and this data was published in the Population Projections for India and States 2011–2036 Report [10]. The population projections for 21 States and one Union Territory in India were calculated using the component method, which applies the assumptions for fertility, mortality, life expectancy and sex ratio at birth.

#### Statistical analysis

Hypertension was defined as a systolic blood pressure (SBP) ≥ 140 mmHg or a diastolic blood pressure (DBP) ≥ 90 mmHg at baseline or participants reported receiving blood pressure-lowering medication. Body mass index (BMI) was calculated as weight in kilograms divided by the square of height in meters, and was categorised as: underweight (< 18.5 kg/m^2^), normal weight (18.5–25.0 kg/m^2^) and overweight (≥ 25.0 kg/m^2^). These definitions for hypertension and assessment of BMI are the same as those used in the NFHS-5 Survey [21].

Categorical variables were reported as frequencies and proportions. Continuous variables were reported as means and standard deviations (SD). The prevalence estimates of hypertension and BMI in the NCDRI Study were calculated overall and by age (adjusted for sex) and sex (adjusted for age). The age-standardized prevalence estimates from the NCDRI Study and the Bihar State NFHS-5 Survey were only estimated for overlapping age groups; hypertension estimates were calculated for women aged 35–49 and men aged 35–54 years and BMI estimates were calculated for women and men aged 40–49 years. The age-standardized prevalence estimates were generated by weighting these estimates to the age distribution of the WHO standard population [22]. To estimate the potential future population burden of NCDs in Bihar, the age and sex-specific estimates of hypertension and BMI were applied to projected population estimates for 2021 and 2036 using data from the 2011 Census of India [10]. All analyses were performed using R (version 4.1.1).

## Results

The baseline characteristics of the participants of the NCDRI Study are presented in Table 1. The study population comprised of 2030 participants including 1005 women. The mean age of the population was 51.3(SD: 10.2) years (49.9 [10.1] women, 52.7 [10.2] men). The mean SBP (122.8 mmHg), DBP (76.2 mmHg), BMI (22.2 kg/m^2^) were similar for men and women, but there was a somewhat higher prevalence of self-reported diabetes among men (5.7% vs 4.9%). Seven out of ten women had no formal schooling (71.1%) compared to only one in four of men (26.4%). Compared to women, men were more likely to report currently being a tobacco smoker (23.3% vs 5.3%), tobacco chewer (53.3% vs 1.7%), and consume alcohol (32.8% vs 0.2%).

**Table 1:** Baseline characteristics of the 2030 participants aged 35–70 years in the NCDRI study

	**Women**	**Men**
Number of participants	1005	1025
Age, years	49.9 (10.1)	52.7 (10.2)
Age categories, years		
35–39	197 (19.6)	112 (10.9)
40–49	315 (31.3)	309 (30.1)
50–59	272 (27.1)	289 (28.2)
60–70	221 (22.0)	315 (30.7)
**Sociodemographic**		
Hindu	990 (98.5)	1002 (97.8)
Highest level of education		
No formal schooling	715 (71.1)	271 (26.4)
1–4 years	151 (15.0)	120 (11.7)
5–8 years	118 (11.7)	402 (39.2)
9 years or more	21 ( 2.1)	232 (22.6)
Occupation		
Housewife/household	712 (70.8)	21 ( 2.0)
Agricultural labour	191 (19.0)	470 (45.9)
Self employed/own business	16 (1.6)	268 (26.1)
Other	86 (8.6)	266 (26.0)
Tap/piped running water connection	460 (45.8)	465 (45.4)
Self-contained toilet	337 (33.5)	422 (41.2)
**Lifestyle factors**		
Current tobacco smoker only	53 ( 5.3)	239 (23.3)
Current tobacco chewer only	17 (1.7)	545 (53.2)
Current drinker of alcohol	2 (0.2)	336 (32.8)
Self-reported diabetes	49 (4.9)	58 (5.7)
**Biological measures**		
SBP, mmHg	121.2 (19.0)	124.3 (18.4)
DBP, mmHg	74.3 (11.7)	78.0 (11.6)
Weight, kg	48.8 (11.3)	58.0 (15.1)
Height, m	1.48 (0.07)	1.61 (0.08)
BMI, kg/m^2^	22.2 (4.9)	22.3 (5.9)

*Abbreviations:* *BMI* body mass index, *DBP* diastolic blood pressure, *SBP* systolic blood pressure

Data are n (%) or mean (SD) Other occupation includes non-agricultural labour, health worker, student, not in labour force, retired, other

The prevalence of hypertension from the NCDRI study is presented in Table 2. Just over one-quarter (27.3% of women and 27.6% of men) in the NCDRI Study had hypertension. The prevalence of hypertension increased with age for all participants, but it was higher in men compared to women at younger ages (20.5% in men and 14.7% in women aged 35–39 years) and higher in women than men at older ages (41.8% in women and 37.3% in men aged 65–70 years). The reported prevalence of hypertension in the NFHS-5 Survey was 8.9% for women aged 15–49 and 12.3% for men aged 15–54 years. The age and sex-specific prevalence estimates of hypertension in the NCDRI Study and the NFHS-5 Survey were comparable in overlapping age groups for women aged 35–49 (19.0% and 17.3%) and men aged 35–54 years (22.9% and 21.5%) (Table 2).

**Table 2:** Age and sex-specific prevalence of hypertension in the NCDRI study and the NFHS-5 survey

**Women**				
**Age (years)**	**NCDRI Study**		**NFHS-5 Survey**	
	**N**	**Hypertension (%)**	**N**	**Hypertension (%)**
** 15–19**			9338	3.2
** 20–24**			7564	4.7
** 25–29**			6293	6
** 30–34**			5250	8.7
** 35–39**	197	14.7	4887	12.2
** 40–44**	168	17.9	3845	17.5
** 45–49**	147	25.2	3800	23.1
** 50–54**	162	24.7		
** 55–59**	110	34.5		
** 60–64**	99	38.4		
** 65–70**	122	41.8		
**Overall, not standardized**	1005	27.3	40,978	8.9
**WHO age standardized at 35–49 years (95%CI)**		19.0(15.4–23.2)		17.3(16.6–18)
**Men**				
**Age (years)**	**NCDRI Study**		**NFHS-5 Survey**	
	**N**	**Hypertension (%)**	**N**	**Hypertension (%)**
** 15–19**			1038	2.6
** 20–24**			705	7.7
** 25–29**			567	8.4
** 30–34**			494	12.6
** 35–39**	112	20.5	518	15.2
** 40–44**	147	17.7	396	19.1
** 45–49**	162	28.4	434	25.5
** 50–54**	170	26.5	329	28.5
** 55–59**	119	35.3		
** 60–64**	130	29.2		
** 65–70**	185	37.3		
** Overall, not standardized**	1025	27.6	4481	12.3
** WHO age standardized at 35–54 years (95%CI)**		22.9(19.2–27.3)		21.5(19.4–23.9)

Hypertension was defined as a SBP ≥ 140 mmHg or DBP ≥ 90 mmHg at baseline or participants reported receiving blood pressure-lowering medication. Hypertension estimates from the NCDRI Study were adjusted for age and sex, where appropriate. Hypertension estimates from the Bihar State NFHS-5 have been previously published [21]. The age-standardized prevalence estimates from the NCDRI Study and the NFHS-5 Survey were only estimated for overlapping age groups, such that hypertension estimates were calculated for women aged 35–49 and men aged 35–54 years. The age-standardized prevalence estimates were generated by weighting these estimates to the age distribution of the WHO’s standard population [22]

In the NCDRI Study, 22.1% of women were underweight (BMI < 18.5 kg/m^2^) and 23.5% were overweight (BMI ≥ 25.0 kg/m^2^) as compared to 17.7% and 20.2% of men (Table 3). The proportion of women who were overweight increased with age to one-quarter of women aged 60–70 (25.8%) while it decreased to one-sixth of men (16.2%) in that age group. The proportion who were underweight was slightly higher in older than younger men (15.2% at 35–39 years vs 21.6% at 60–70 years). The NFHS-5 Survey reported that 25.6% of women aged 15–49 years were underweight and 15.9% were overweight versus 21.5% and 14.7% of men. For men aged 50–54 years, in the NCDRI study 16.5% were underweight and 21.8% were overweight, and in the NFHS-5 survey the corresponding estimates were 13.3% and 24.1%, with similar age-standardized proportions. BMI estimates are reported in ten year age groupings in the NFHS-5 survey for all other age-groups among men and women, and we compared BMI categories among those aged 40–49 years across the NCDRI Study and the NFHS-5 Survey in this overlapping age group, and these age-standardized proportions were similar (Table 3).

**Table 3:** The age and sex-specific distribution of BMI in the NCDRI study and the NFHS-5 survey

**Women**								
	**NCDRI Study**				**NFHS-5 Survey**			
**Age (years)**	**N**	**Underweight** **(< 18.5 kg/m**^**2**^**)**	**Normal weight (18.5–25.0 kg/m**^**2**^**)**	**Overweight** **(≥ 25.0 kg/m**^**2**^**)**	**N**	**Underweight** **(< 18.5 kg/m**^**2**^**)**	**Normal weight (18.5–25.0 kg/m**^**2**^**)**	**Overweight** **(≥ 25.0 kg/m**^**2**^**)**
** 15–19**					8818	43.6	53.8	2.6
** 20–29**					11,610	27	62.1	10.9
** 30–39**	197	24.9	54.8	20.3	9779	16.4	59.4	24.2
** 40–49**	315	20.6	54.0	25.4	7639	14.2	57.3	28.5
** 50–59**	272	18.8	59.6	21.7				
** 60–70**	221	25.8	48.4	25.8				
**Overall, not standardized**	1005	22.1	54.4	23.5	37,846	25.6	58.5	15.9
**WHO age standardized at 40–49 years (95%CI)**		20.6(13.3–29.8)	54.0(46.2–62.8)	25.4(17.9–34.5)		14.2(12.6–16.0)	57.3(55.6–59.0)	28.5(26.8–30.2)
**Men**								
	**NCDRI Study**				**NFHS-5 Survey**			
**Age (years)**	**N**	**Underweight** **(< 18.5 kg/m**^**2**^)	**Normal weight (18.5–25.0 kg/m**^**2**^**)**	**Overweight** **(≥ 25.0 kg/m**^**2**^**)**	**N**	**Underweight (< 18.5 kg/m**^**2**^**)**	**Normal weight (18.5–25.0 kg/m**^**2**^**)**	**Overweight** **(≥ 25.0 kg/m**^**2**^**)**
** 15–19**					1036	46.2	51.4	2.4
** 20–29**					1272	16.6	72.5	10.9
** 30–39**	112	15.2	58.0	26.8	1011	11.6	65.6	22.8
** 40–49**	309	13.6	67.6	18.8	827	10.4	63.8	25.8
** 50–59**	289	18.7	57.8	23.5				
** 60–70**	315	21.6	62.2	16.2				
**Overall, not standardized**	1025	17.7	62.1	20.2	4146	21.5	63.8	14.7
**WHO age standardized at 40–49 years (95%CI)**		13.6(6.0–24.5)	67.6(58.7–77.4)	18.8(10.8–29.3)		10.4(5.7–16.6)	63.8(58.5–69.5)	25.8(20.6–31.6)

BMI was calculated as weight in kilograms divided by the square of height in meters, and it was further categorized as: underweight (< 18.5 kg/m^2^), normal weight (18.5–25.0 kg/m^2^) and overweight (≥ 25.0 kg/m^2^). BMI estimates from the NCDRI Study were adjusted for age and sex, where appropriate. BMI estimates from the Bihar State NFHS-5 have been previously published [21]. The age-standardized prevalence estimates from the NCDRI Study and the NFHS-5 Survey were only estimated for overlapping age groups, such that BMI estimates were calculated for women and men aged 40–49. The age-standardized prevalence estimates were generated by weighting these estimates to the age distribution of the WHO’s standard population [22]

**Fig. 1:**
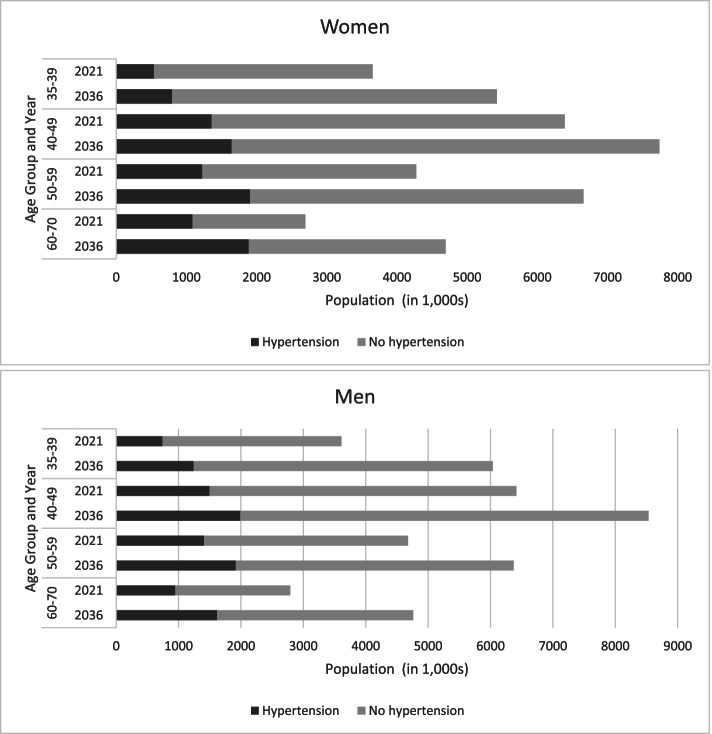
Estimates of hypertension by sex and year for Bihar India using the age-specific estimates of hypertension from the NCDRI Study and population estimates from the 2011 Census of India. Hypertension was defined as a SBP ≥ 140 mmHg or DBP ≥ 90 mmHg at baseline or participants reported receiving blood pressure-lowering medication. The age and sex-specific estimates of hypertension obtained from the NCDRI Study were applied to projected population estimates for 2021 and 2036 using data from the 2011 Census of India Population Projections for India and States 2011–2036 Report [10]

**Fig. 2:**
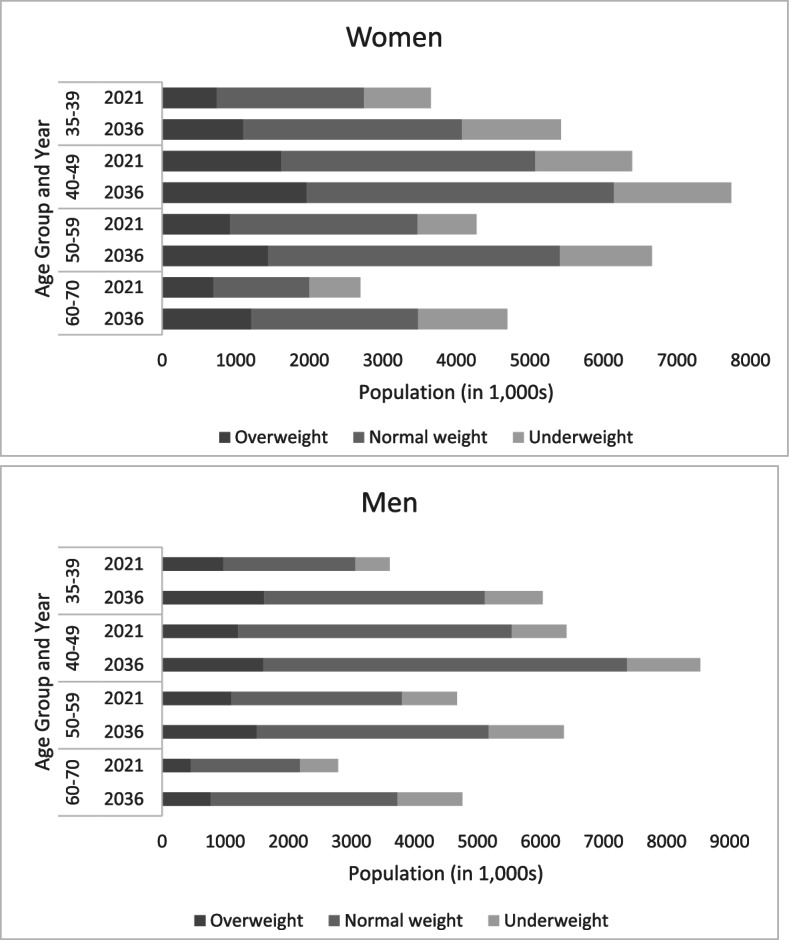
Estimates of underweight, normal weight and overweight adults by sex and year in Bihar, India using the age-specific estimates of BMI from the NCDRI Study and population estimates from the 2011 Census of India. BMI was calculated as weight in kilograms divided by the square of height in meters, and it was further categorized as: underweight (< 18.5 kg/m^2^), normal weight (18.5–25.0 kg/m^2^) and overweight (≥ 25.0 kg/m^2^). The age and sex-specific estimates BMI obtained from the NCDRI Study were applied to projected population estimates for 2021 and 2036 using data from the 2011 Census of India Population Projections for India and States 2011–2036 Report [10]

Data from the 2011 Census of India was used to estimate the population of India nationally and at state level in 2021 and 2036 [10]. In 2021, the population of Bihar was estimated to be 123 million. Of these, 28% were aged 35–69 years and this is expected to increase to 34% in 2036 with a roughly equal distribution of women and men (Additional file: Supplementary Fig. 1), and the number of people in the 60–64 age group will nearly double. Estimated prevalence of hypertension and BMI categories in Bihar for 2021 and 2036 are shown in Figs. 1 and 2 (Additional file: Supplementary Tables 2 and 3). In 2021, we estimated that approximately 9 million adults aged 35–69 years had hypertension in 2021 and this will increase to 14 million in 2036. When looking at weight, we estimated that 7 million adults were underweight and 8 million were overweight in 2021and this is expected to increase to 8 million underweight and 9 million overweight adults in 2036.

## Discussion

In this study of men and women aged 35–70 years, the prevalence of both hypertension and overweight were higher than in the contemporaneous NFHS-5 survey of men aged 15–54 years and women aged 15–49 years. However, where age groups overlapped, the age-standardized estimates were similar for both hypertension and BMI categories between the NCDRI Study and the NHFS-5 Survey. These results suggest that there is a growing burden of NCDs among older adults in rural India, which is not being captured by current routine surveys.

The estimates from the NCDRI survey are similar to other national studies in India [5, 23–25]. For example, Geldsetzer et al. (2018) reported that the crude prevalence of hypertension in India was 25.3% (95% CI: 25.0–25.6) among adults aged 18 years and older, comparable to a prevalence in rural Bihar of 24.0% (95% CI: 21.3–26.7) [23]. Higher estimates have been reported in a study of adults aged 45 years and older, 41.9% of adults and their spouses had hypertension compared with an overall estimate of 35.3% in the State of Bihar [24]. One-fifth of women and one-quarter of men reported being overweight in the NCDRI Study, the proportion of women who were overweight appeared to increase with age while the opposite trend was observed for men. These estimates are similar to other studies showing that the rates of obesity are increasing in rural areas; however, there is a high degree of variation of obesity estimates within India [26–28].

The observed differences between the NCDRI Study and the NFHS-5 Survey are likely to reflect the differences in the age and sex distribution of participants. In the NFHS-5 Survey for Bihar, the oldest participant was 54 years for men and 49 years for women, and just 10% of participants were men [21], underrepresenting men and older people [10]. Furthermore, the lower reported prevalence of overweight adults in the NFHS-5 Survey may be due to the fact that the Bihar estimates are state-level and may not reflect the geographic variation of BMI in urban and rural settings [26]. For example, the NFHS-5 Survey from the district of Patna, Bihar, where the NCDRI Study was conducted, reported that 22.6% of women were underweight and 21.5% were overweight [29], which is similar to the NCDRI Study. The district level estimates for the prevalence of hypertension were much lower than in our study, at 14.8% for men and 16.1% for women, and this is likely to reflect the younger age distribution of the NHFS-5 survey, as increasing incidence of hypertension is associated with increasing age [30].

The underestimation of NCDs in national surveys has long-term implications because the burden of NCDs will continue to grow as the population of India ages. Using the NCDRI disease estimates and the state level population projections, the expected number of adults aged 35–69 years in Bihar with hypertension or who are overweight will increase from 2021 to 2036 and most substantially among those aged 60–69 years (3.5 million with hypertension and 1.7 million overweight). Underestimation of these major causes of NCDs in older age groups could have a potentially large impact on the allocation of healthcare resources and could lead to greater unmet medical needs. Recently, there has been a widespread reform of the primary healthcare system in rural India, driven by the increasing recognition that the burden of disease has shifted from infectious diseases to chronic comorbidities and that more than half of these conditions can be managed appropriately at the primary care level [31–33]. These policies address disease control and management through the delivery of programmes aimed at prevention through modification of known risk factors, early detection and adequate treatment, in order to reduce disability and mortality from NCDs at much lower costs.

One of the strengths of the NCDRI Study is the near equal participation by sex and the broader range of ages included to generate a representative population for where the risk factors of interest are becoming more common. The cross-sectional study design has some limitations. Self-reported medical history may be subject to recall bias, resulting in an over- or underestimation of the prevalence of NCDs. However, in this analysis, the self-reported estimates of hypertension were coupled with the standardised measures of SBP and DBP[19]. Blood pressure measurements were recorded on a single visit only which can potentially overestimate the prevalence of hypertension, but this methodology has been validated by the WHO STEPS methodology [19], and is also comparable to the methodology employed for the NFHS-5 Survey. The age and sex-specific estimates of hypertension and BMI were extrapolated from the rural NCDRI Study to calculate projected trends of NCDs in 2021 and 2036, and therefore, these estimates may not account for the changing population at risk over time, or interventions of control and prevention. Although Bihar is a largely rural state, we used prevalence estimates from only a rural setting and as such, any important variations between urban and rural prevalence rates will not be accounted for when estimating the future projected trends.

The results from the NCDRI study suggest that state-level data from routine surveys in rural India does not fully capture the burden of NCDs in older age groups, a population that can experience a significant burden of disease. Other surveys, such as the NNMS, where older individuals are sampled also have limitations by providing national level aggregate data, and could provide more granular estimates by age and region, which is currently lacking [16]. A generally higher prevalence of hypertension and obesity in older age groups in rural India is likely to lead to an increased risk of comorbidities and premature mortality, as well as creating a strain on the health care system [34]. To account for changing demographics as the population of India ages, future health surveys should widen the age range of participants and have equal participation of women and men, in order to provide more accurate estimates of NCDs and their risk factors, and to help inform healthcare planning that is relevant to local need in underserved rural areas.

## Supplementary Information

**Additional file 1: Table 1.** Inclusion criteria for the five iterations of National Family Health Surveys in India. **Table 2.** Estimates of hypertension in Bihar India for 2021 and 2036 using the age-specific estimates of hypertension from the NCDRI Study and population estimates from the 2011 Census of India. **Table 3.** Estimates of BMI in Bihar India for 2021 and 2036 using the age-specific estimates of BMI from the NCDRI Study and population estimates from the 2011 Census of India. **Figure 1.** Population pyramid for Bihar India for men and women in 2021 and 2036 using population estimates from the 2011 Census of India.

## Data Availability

The data that support the findings of this study are available from the corresponding author (TG) upon reasonable request.
