# Supplementary material for: The burden of risk factors for non-communicable disease in rural Bihar, India: a comparative study with national health surveys
Source: BMC Public Health. 2022 Aug 12;22:1538. doi: 10.1186/s12889-022-13818-1 (PMC9375264; doi:10.1186/s12889-022-13818-1)
Supplement: Supplementary file 1 — Additional file 1: Table 1. Inclusion criteria for the five iterations of National Family Health Surveys in India. Table 2. Estimates of hypertension in Bihar India for 2021 and 2036 using the age-specific estimates of hypertension from the NCDRI Study and population estimates from the 2011 Census of India. Table 3. Estimates of BMI in Bihar India for 2021 and 2036 using the age-specific estimates of BMI from the NCDRI Study and population estimates from the 2011 Census of India. Figure 1. Population pyramid for Bihar India for men and women in 2021 and 2036 using population estimates from the 2011 Census of India. [file 12889_2022_13818_MOESM1_ESM.pdf]

Supplementary Table 1: Inclusion criteria for the five iterations of National Family Health Surveys in India.

Iteration	Year	Women		Men	
		Age (years)	N	Age (years)	N
1	1992-93	Ever married 13-49	89,777	NA	
2	1998-99	Ever married 15-49	89,111	NA	
3	2005-06	15-49	124,385	15-54	74,369
4	2015-16	15-49	699,686	15-54	112,122
5	2019-20	15-49	724,115	15-54	101,839

Abbreviation: NA = not available. Previously published data was obtained from the NFHS-1, NFHS-2, NFHS-3, NFHS-4 and NFHS-5[11-15].

Supplementary Table 2: Estimates of hypertension in Bihar India for 2021 and 2036 using the age-specific estimates of hypertension from the NCDRI Study and population estimates from the 2011 Census of India.

Group	Age (years)	Population (N)		Hypertension (N)	
		2021	2036	2021	2036
All	35-39	7,266,000	11,462,000	1,282,951	2,023,835
	40-49	12,807,000	16,275,000	2,855,373	3,628,578
	50-59	8,955,000	13,032,000	2,632,517	3,831,039
	60-70	5,488,000	9,455,000	2,035,435	3,506,748
	All	34,516,000	50,224,000	9,385,632	13,656,969
Women	35-39	3,655,000	5,426,000	538,046	798,751
	40-49	6,392,000	7,740,000	1,359,568	1,646,286
	50-59	4,276,000	6,660,000	1,226,206	1,909,853
	60-70	2,697,000	4,694,000	1,086,122	1,890,344
	All	17,020,000	24,520,000	4,652,759	6,703,035
Men	35-39	3,611,000	6,036,000	741,545	1,239,536
	40-49	6,415,000	8,535,000	1,494,757	1,988,738
	50-59	4,679,000	6,372,000	1,408,557	1,918,215
	60-70	2,791,000	4,761,000	948,054	1,617,229
	All	17,496,000	25,704,000	4,824,807	7,088,297

Hypertension was defined as a SBP ≥140 mmHg or DBP ≥ 90 mmHg at baseline or a diagnosis of hypertension and were receiving blood pressure-lowering medication. The age and sex-specific estimates of hypertension obtained from the NCDRI Study were applied to projected population estimates for 2021 and 2036 using data from the 2011 Census of India Population Projections for India and States 2011–2036 Report[10].

Supplementary Table 3: Estimates of BMI in Bihar India for 2021 and 2036 using the age-specific estimates of BMI from the NCDRI Study and population estimates from the 2011 Census of India.

Group	Age (years)	Population (N)		Underweight (<18.5 kg/m2) (N)		Normal weight (18.5-25.0 kg/m2) (N)		Overweight (≥25.0 kg/m2) (N)	
		2021	2036	2021	2036	2021	2036	2021	2036
All	35-39	7,266,000	11,462,000	1,551,961	2,448,194	4,068,020	6,417,237	1,646,019	2,596,569
	40-49	12,807,000	16,275,000	2,196,073	1,744,409	7,778,611	6,178,793	2,832,317	2,249,798
	50-59	8,955,000	13,032,000	1,676,070	1,664,278	5,251,685	5,214,738	2,027,246	2,012,984
	60-70	5,488,000	9,455,000	1,279,851	1,949,161	3,102,358	4,724,765	1,105,791	1,684,074
	All	34,516,000	50,224,000	6,703,954	7,806,042	20,200,673	22,535,533	7,611,373	8,543,426
Women	35-39	3,655,000	5,426,000	909,112	1,349,614	2,003,757	2,974,660	742,132	1,101,726
	40-49	6,392,000	7,740,000	1,318,984	1,597,143	3,449,651	4,177,143	1,623,365	1,965,715
	50-59	4,276,000	6,660,000	801,750	1,248,750	2,546,735	3,966,617	927,515	1,444,633
	60-70	2,697,000	4,694,000	695,606	1,210,670	1,305,787	2,272,661	695,606	1,210,670
	All	17,020,000	24,520,000	3,725,452	5,406,177	9,305,930	13,391,081	3,988,619	5,722,743
Men	35-39	3,611,000	6,036,000	548,098	916,178	2,095,669	3,503,035	967,232	1,616,785
	40-49	6,415,000	8,535,000	871,942	1,160,097	4,338,948	5,772,864	1,204,110	1,602,039
	50-59	4,679,000	6,372,000	874,277	1,190,616	2,703,782	3,682,090	1,100,941	1,499,294
	60-70	2,791,000	4,761,000	602,502	1,027,771	1,736,622	2,962,400	451,876	770,829
	All	17,496,000	25,704,000	2,896,818	4,294,663	10,875,022	15,920,390	3,724,160	5,488,947

BMI was calculated as weight in kilograms divided by the square of height in meters, and it was further categorized as: underweight (<18.5 kg/m^2), normal weight (18.5-25.0 kg/m^2) and overweight (≥ 25.0 kg/m^2). The age and sex-specific estimates BMI obtained from the NCDRI Study were applied to projected population estimates for 2021 and 2036 using data from the 2011 Census of India Population Projections for India and States 2011–2036 Report[10].

Supplementary Figure 1: Population pyramid for Bihar India for men and women in 2021 and 2036 using population estimates from the 2011 Census of India.

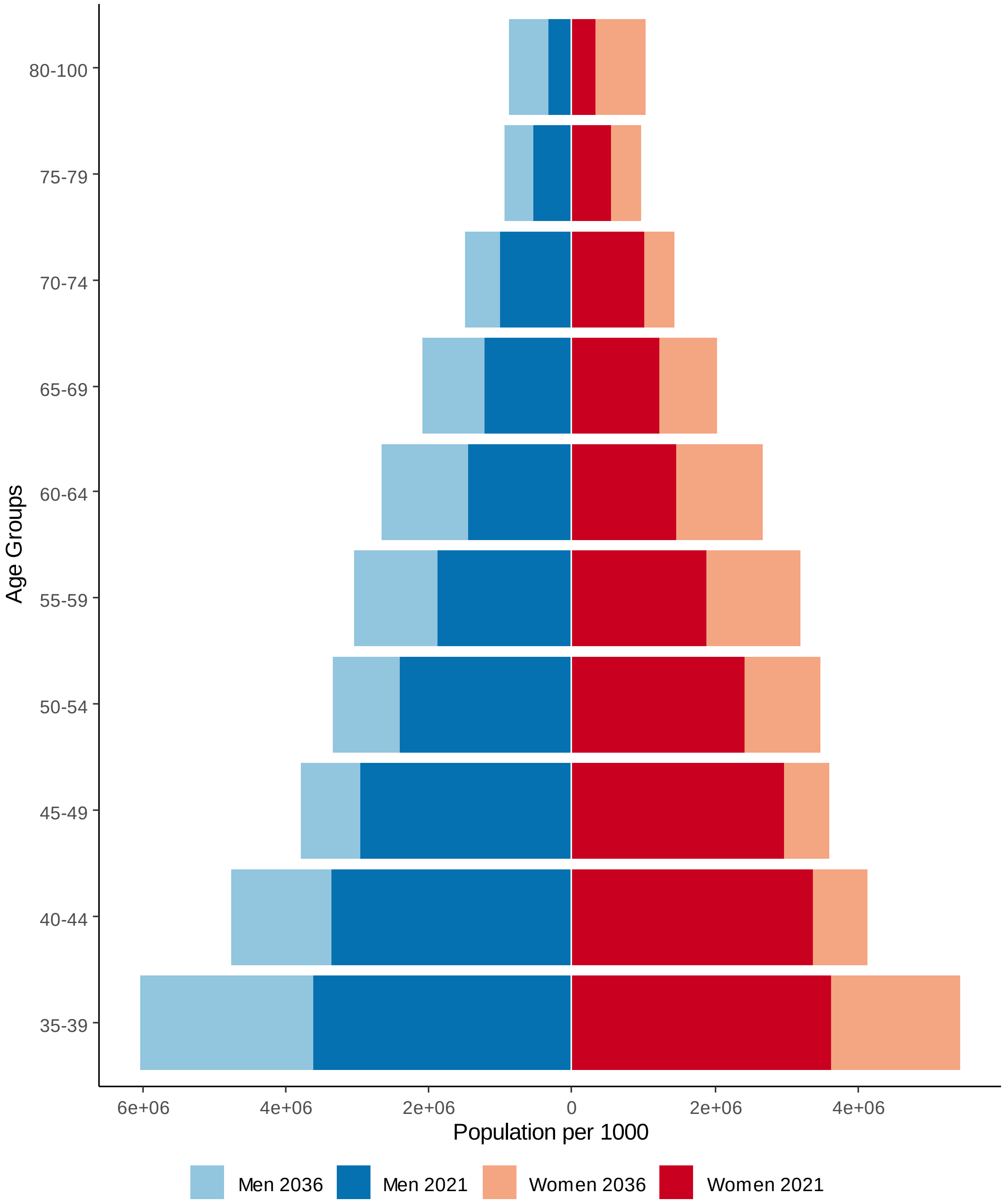

Projected population estimates for 2021 and 2036 were obtained from the 2011 Census of India Population Projections for India and States 2011–2036 Report[10].
